# Concurrent Ingestion of Alkaline Water and L-Glutamine Enhanced Salivary α-Amylase Activity and Testosterone Concentration in Boxing Athletes

**DOI:** 10.3390/nu16030454

**Published:** 2024-02-05

**Authors:** Tung-Lin Lu, Cheng-Shiun He, Katsuhiko Suzuki, Chi-Cheng Lu, Chung-Yuan Wang, Shih-Hua Fang

**Affiliations:** 1 Institute of Athletics, National Taiwan University of Sport, Taichung 404401, Taiwan; 11004012@gm.ntus.edu.tw (T.-L.L.); a722353@ntus.edu.tw (C.-C.L.); 2 Department of Athletic Sports, National Chung Cheng University, Minxiong 621301, Taiwan; cshe@ccu.edu.tw; 3 Faculty of Sport Sciences, Waseda University, Tokorozawa 359-1192, Japan; katsu.suzu@waseda.jp; 4 Department of Combat Sports, National Taiwan University of Sport, Taichung 404401, Taiwan; cywang@ntus.edu.tw

**Keywords:** alkaline water, L-glutamine, salivary immunity, hormone, exercise recovery

## Abstract

Athletes often take sport supplements to reduce fatigue and immune disturbances during or after training. This study evaluated the acute effects of concurrent ingestion of alkaline water and L-glutamine on the salivary immunity and hormone responses of boxers after training. Twelve male boxing athletes were recruited in this study. During regular training, the participants were randomly divided into three groups and asked to consume 400 mL of alkaline water (Group A), 0.15 g/kg body weight of L-glutamine with 400 mL of water (Group G), and 0.15 g/kg of L-glutamine with 400 mL of alkaline water (Group A+G) at the same time each day for three consecutive weeks. Before and immediately after the training, saliva, heart rates, and the rate of perceived exertion were investigated. The activity of α-amylase and concentrations of lactoferrin, immunoglobulin A (IgA), testosterone, and cortisol in saliva were measured. The results showed that the ratio of α-amylase activity/total protein (TP) significantly increased after training in Group A+G but not in Group A or G, whereas the ratios of lactoferrin/TP and IgA/TP were unaffected in all three groups. The concentrations of salivary testosterone after training increased significantly in Group A+G but not in Group A or G, whereas the salivary cortisol concentrations were unaltered in all groups. In conclusion, concurrent ingestion of 400 mL of alkaline water and 0.15 g/kg of L-glutamine before training enhanced the salivary α-amylase activity and testosterone concentration of boxers, which would be beneficial for post-exercise recovery.

## 1. Introduction

Boxing is one of the oldest combat sports. Well-developed muscle strength, muscle power, and anaerobic power and capacity are key components to success in boxing given its high-intensity and intermittent nature [1,2]. During repeated bouts of high-intensity exercise, H^+^ is produced by anaerobic metabolism [3]. Intramuscular H^+^ accumulation causes muscle acidosis and impairs energy transfer, calcium handling, and cross-bridge cycling, thereby limiting performance during high-intensity exercise [4]. Metabolic acidosis occurs in boxers after sparring for four rounds of 3 min separated by 1 min of rest, thereby affecting punch efficacy [5].

Athletes participating in prolonged heavy intensive exercise may exhibit immune impairment that makes it easier for viruses to invade, and infections may occur during the period referred to as “the open window” [6,7]. Saliva mucosal secretions and antimicrobial proteins provide the first line of defense against pathogens at mucosal surfaces [8]. The antimicrobial proteins in saliva, such as lactoferrin and immunoglobulin A (IgA), play important roles in human innate defense against bacteria, fungi, and viruses [9,10,11]. Our previous study revealed that the salivary immune markers of athletes were modulated during training and competition periods [12]. Basketball players’ secretion rates and concentrations of salivary IgA and lactoferrin were significantly decreased during a 4 week program of intensive training and six competition periods [13]. In combat sports, high-intensity training and rapid weight reduction affected the mucosal immunity [14]. The four-week pre-competition training program and rapid weight reduction impaired the mucosal immunity and increased the incidence of upper respiratory tract infection in elite male taekwondo athletes after competition [15].

Salivary hormones such as testosterone and cortisol are known as markers of anabolic/catabolic status, overtraining, and training responses [16]. Testosterone represents the body’s anabolic state and induces skeletal muscle hypertrophy by multiple mechanisms, including its effects on modulating the commitment of pluripotent mesenchymal cells, leading to improved muscle strength and power [17]. Soccer players who exhibited better physical performance after 6 weeks of training showed higher testosterone concentrations [18]. The stress hormone cortisol stimulates lipolysis in adipose cells and decreases protein synthesis in muscle cells [19]. Physical stress such as exercise, increases circulating cortisol, which is influenced by the intensity and duration of exercise [20,21]. To monitor whether an athlete is at risk of overtraining, the postexercise hormone response is a more reliable biomarker than resting levels [20,22].

One of the most important strategies to minimize fatigue and immune disturbance during and after training is the use of sports supplements [23,24,25]. A previous study reported that treadmill and ergometer exercises decrease salivary buffering capacity, and that drinking water during exercise can prevent excessive dehydration and changes in electrolyte balance while maintaining salivary secretion function [26]. Alkaline water, a nutritional aid for lowering acidity with antioxidant and antiaging properties, has demonstrated its effectiveness as an alkalizing agent in the treatment of metabolic acidosis in both animal and human research [27,28]. Past studies have shown that daily intake of 2.5–4 L of alkaline water for 3~6 weeks has significant impacts on anaerobic performance and acid–base balance in combat sport and basketball athletes [28,29].

Glutamine is the most abundant amino acid in the body. Immune cells such as lymphocytes, neutrophils, and macrophages utilize glutamine at high rates under catabolic conditions such as high-intensity/volume physical exercise [30]. Thus, glutamine is an essential nutrient for the immune system. Plasma glutamine concentrations decreased significantly in runners who participated in 9–9.5 days of twice-a-day interval training [31]. Recreationally active males demonstrated significantly decreased glutamine concentrations following strenuous interval training sessions [32]. Reduced glutamine levels are responsible for the immune suppression associated with increased infection rates observed in overtrained athletes [33]. A previous study suggested that pre-exercise ingestion of 0.3 g/kg/day L-glutamine for 3 days could maintain its physiological concentration during exercise, which might speed up muscle recovery with less muscle damage [34].

Alkaline water and L-glutamine both have the benefits of maintaining athletes’ performance and enhancing recovery during and after high-intensity exercise. Previous studies that combined different nutritional supplements have shown some beneficial results [35,36]. Until now, no studies have combined alkaline water and L-glutamine to determine whether the combination is more beneficial for athletes. Therefore, this study assessed the acute effects of co-supplementation of alkaline water and L-glutamine on the salivary immunity and hormone responses of boxers after training.

## 2. Materials and Methods

### 2.1. Participants

The characteristics of the participants are presented in Table 1. Twelve male boxing athletes from National Taiwan University of Sport were recruited in this study. The subjects were excluded if they were injured or were unable to participate in regular training. All participants were non-smokers and advised to refrain from strenuous exercise, caffeine, and alcohol consumption before the study. We requested the participants to maintain consistent dietary choices throughout the study period. Each participant was fully informed of all potential risks and experimental procedures, after which informed written consent was obtained. All experimental procedures and protocols were approved by the Institutional Human Ethics Committee of Jen-Ai Hospital, Taichung, Taiwan (Approval No. #108-85).

### 2.2. Study Design

This study was conducted in a randomized and crossover manner. The participants continued their regular 2 h boxing training during the study. The training session comprised specific training and sparring techniques. The athletes’ physical activities during 2 h boxing training were determined using an ActiGraph GT3X activity monitor (ActiGraph, LLC., Pensacola, FL, USA). During training, the participants were asked to consume 400 mL of alkaline water (Group A), 0.15 g/kg bw (body weight) of L-glutamine with 400 mL of water (Group G), or 0.15 g/kg bw of L-glutamine with 400 mL of alkaline water (Group A+G) in a randomized order at the same time each day for 3 consecutive weeks. The block randomization method was used to randomly divide the participants to afford an equal sample size in each group [37]. The selected dose of L-glutamine (0.15 g/kg body weight) was determined by referring to previous studies using a dose range at 0.05–0.30 g/kg body weight [38,39], and the practical ingestion of the glutamine powder by the athletes was taken into consideration. The training session was held from 1 pm to 3 pm. At 30 min before 1 pm and immediately after the training session, saliva, heart rate, and rate of perceived exertion (RPE) were analyzed. Two milliliters of saliva samples were collected into sterile plastic containers and stored at −80 °C until analysis. The athlete’s heart rate was measured using a Rossmax SB100 fingertip pulse oximeter (Rossmax International Ltd., Taipei, Taiwan), and the Borg Category-Ratio-10 Scale [40] was used to assess the RPE of the athlete. All the experiments were conducted at the same time of day. After supplementation, the participants were allowed to drink water ad libitum during the training session.

### 2.3. Analysis of Salivary Proteins

The bovine serum albumin (BSA) protein was detected by using the BioRAD protein assay kit (Bio-RAD, Hercules, CA, USA) to represent the total protein (TP) content. The salivary α-amylase activity was determined using a kinetic reaction assay kit (Salimetrics LLC., State College, PA, USA). Both methods were used by following the manufacturer’s instructions [41,42]. The concentrations of salivary lactoferrin, IgA, testosterone, and cortisol were measured using enzyme-linked immunosorbent assays (ELISA, Calbiochem, Darmstadt, Germany) following the manufacturer’s instructions. All samples were analyzed in duplicate. The intra-assay coefficient of variation (CV) was 2% for salivary α-amylase, IgA, and lactoferrin, 1% for salivary cortisol, and 0.3% for salivary testosterone.

### 2.4. Statistical Analysis

The results are expressed as the mean ± standard deviation (SD). The Shapiro–Wilk test was used to analyze the distribution of datasets. The estimated sample size to identify differences in mucosal immunity after exercise was nine subjects based on an alpha level of 0.05 and a beta level of 0.8 [43]. We confirmed the appropriateness of the sample size using the following parameters: a significance level (α) of 0.05, a statistical power level of 0.8, and an effect size of 0.72. The sample size calculation was performed using G*power version 3.1.9.7 software (available at www.gpower.hhu.de (accessed on 6 December 2023), which yielded a total required sample size of 12. The difference in physical activity among the groups during boxing training was tested by one-way analysis of variance (ANOVA). A two-way repeated-measures ANOVA was used to test for differences in the heart rate, rate of perceived exertion and salivary variables. Post hoc analyses were conducted by paired *t*-tests to follow up on statistically significant interactions or main effects. Significant differences were set at * *p* < 0.05 and *** *p* < 0.001, respectively.

## 3. Results

### 3.1. Effects of Co-Supplementation with Alkaline Water and L-Glutamine on Heart Rate and Rate of Perceived Exertion during Boxing Training

The boxing athletes of Groups A, G, and A+G experienced similar physical intensities of training (Table 2). While the heart rates and rates of perceived exertion of the participants in each group were significantly increased after training (*p* < 0.001, Table 2), no differences were noted among the three groups.

### 3.2. Effects of Co-Supplementation with Alkaline Water and L-Glutamine on Changes in Salivary Immune-Related Proteins after Boxing Training

The ratio of salivary α-amylase activity/TP was significantly increased after training in Group A+G (*p* < 0.05, Table 3) but not in Groups A or G. The concentrations of salivary lactoferrin/TP and IgA/TP were not significantly affected in any of the three groups (Table 3).

### 3.3. Effects of Co-Supplementation with Alkaline Water and L-Glutamine on Changes in Salivary Hormones after Training

**Figure 1:**
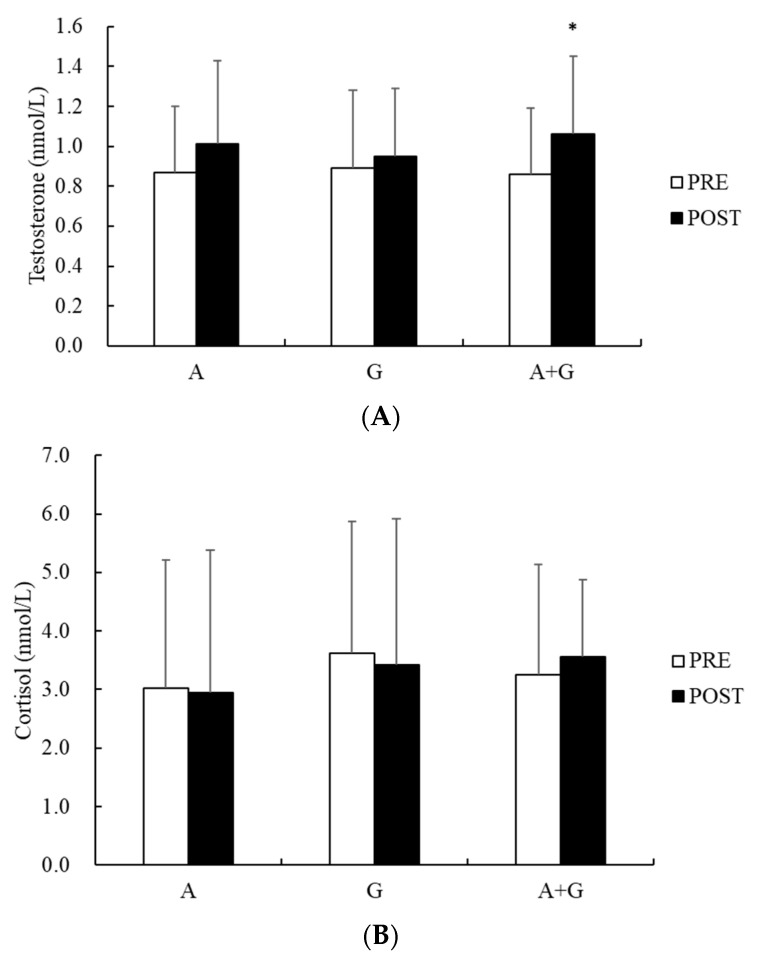
Acute effects of on salivary hormone concentrations ((**A**) testosterone and (**B**) cortisol) after training. Values are mean ± SD. * *p* < 0.05, significantly different from PRE in each group. A: alkaline water group; G: L-glutamine group; A+G: alkaline water + L-glutamine group.

The concentration of salivary testosterone after training was significantly increased in Group A+G (from 0.86 ± 0.33 nmol/L to 1.06 ± 0.39 nmol/L, *p* < 0.05, Figure 1) but not in Groups A or G. However, the salivary cortisol concentration remained unchanged in each group.

## 4. Discussion

This was the first study to investigate the acute effects of co-supplementation with alkaline water and L-glutamine on the immune and hormone responses in boxers. The results showed that co-ingestion of 400 mL alkaline water and 0.15 g/kg L-glutamine increased salivary α-amylase activity and testosterone concentrations after boxing training. No previous study has assessed the effect of alkaline water or L-glutamine on salivary immune markers and hormones. Our present study found that co-ingestion of these two supplements increased the salivary α-amylase activity and testosterone concentration after training, indicating an additive effect of these two supplements. Although we could not explain the exact mechanism for the result of this study, we speculate that these supplements modulated fatigue and had antioxidant and anti-inflammatory effects during and after training, thereby influencing the athletes’ salivary immunity and hormone concentration.

Prolonged exercise and periods of heavy training are associated with a decrease of plasma glutamine concentration, which has been suggested as a potential cause of exercise-induced immune impairment and increased susceptibility to infection in athletes [44]. L-glutamine supplementation improves the body’s immune system in acute stress situations by fueling the immune cells and reducing pro-inflammatory cytokines [45,46]. Chronic supplementation with L-glutamine has shown beneficial effects for recovery after exercise in athletes and elderly subjects. Professional basketball players supplemented with 40 days of L-glutamine displayed significantly low values of creatine kinase and myoglobin in the blood, suggesting less muscle damage compared to placebo [47]. Thirty days of L-glutamine supplementation yielded a better inflammatory response and redox balance in elderly practitioners of combined-exercise training at moderate intensity [48]. In addition, L-glutamine improved the strength and power of knee muscles and glycemic control while boosting plasma antioxidant capacity in exercising elderly women [49]. Based on the known effects of L-glutamine on muscle recovery and immune response, we speculated that it affected the salivary immune markers and hormones in athletes after training.

Regarding the effects of acute supplementation, a previous study investigating co-supplementation with L-glutamine and L-cystine found beneficial results on energy metabolism and fatigue following endurance exercise [50]. Acute oral supplementation of 0.087 g/kg L-leucine plus 0.3 g/kg L-glutamine increased the rate of recovery in isometric strength, counter-movement jump (CMJ) height, delayed-onset muscle soreness (DOMS), and creatine kinase (CK) compared to placebo after eccentrically biased exercise [51]. Soccer players exhibited improvements in time and distance and reported reduced feelings of fatigue after supplementation with 3.5 g L-glutamine plus 50 g maltodextrin 30 min before exercise [38]. Co-ingestion of 0.25 g/kg L-glutamine in 250 mL of water and 50 g maltodextrin 2 h before exercise more effectively prevented the anaerobic power decrease noted in the repeated running-based anaerobic sprint test (RAST) with intervals [52]. Consistent with previous studies, the present study found that co-ingestion of alkaline water and L-glutamine increased athletes’ salivary α-amylase activity, which is beneficial for athletes’ mucosal immunity. Improved immune status can prevent infection in athletes during condensed training schedules and reduce the occurrence of overtraining.

Salivary α-amylase is one of the major enzymes in the oral cavity. Increased salivary α-amylase activity is beneficial to defense against bacteria. Conversely, low salivary α-amylase activity is related to a higher risk of oral infection [53]. L-glutamine increases amylase production by increasing the bacterial cell population, potentially due to the effects of L-glutamine’s nutritional properties as well as its role as a precursor in amino acid synthesis [54,55]. Salivary IgA has the capacity to inhibit the colonization of pathogens, bind antigens for transport across the epithelial barrier, and neutralize viruses, representing one of the body’s first lines of defense against infections related to upper respiratory tract infection [56]. Supplementation with 0.3 g/kg/day L-glutamine for 30 days modulated the salivary cytokine profile and increased salivary IgA levels both total and specific to the influenza virus vaccine in physically active elderly subjects [57]. In contrast to previous studies, we speculate that short-term or acute doses of L-glutamine supplementation might not be sufficient to regulate the activity or concentration of salivary immune markers.

In this study, we found that a single dose of L-glutamine supplementation might not be sufficient to modulate the concentration of salivary immune markers such as IgA and lactoferrin after training. Consistent with our results, strenuous exercise associated with hypoxia with or without L-glutamine supplementation for 6 days did not change the salivary IgA concentration [58]. Runners supplemented with 0.4 g/kg/day of L-glutamine for 14 days exhibited increased nasal but not salivary IgA concentrations [31]. In another study, acute L-glutamine supplementation was not able to abolish the decline in salivary IgA concentration [59]. Until now, there were no studies investigating the change of salivary α-amylase activity after exercise in athletes supplemented with L-glutamine. The present study found that the salivary α-amylase activity of boxing athletes supplemented with a single dose of L-glutamine was not increased after training.

Previously, the mechanism behind the increase in salivary α-amylase, but not lactoferrin and IgA in athletes after co-ingestion of alkaline water and L-glutamine in athletes remained unknown. A significant increase in salivary α-amylase was reported in Taekwondo athletes after an official competition in which the athletes spent 65% of the time working at greater than 90% of the maximum heart rate of each individual [60]. On the contrary, such high-intensity events decreased the level of immunoglobulin, indicating that IgA tends to exhibit low levels or remain unchanged after exhaustive exercises or high-intensity training sessions [61]. Our results indicate that co-ingestion of alkaline water and L-glutamine can significantly elevate the level of salivary α-amylase and potentially prevent the decline in IgA after high-intensity exercise.

Lactoferrin has been shown to increase immediately after exercise with supplementation such as green tea [62]. However, in our study we did not observe any changes after co-ingestion of alkaline water and L-glutamine. Exploring different dosages and timings of supplementation is necessary to determine whether these interventions can aid athletes in preserving their immune function during training and competition.

As to the effects on salivary testosterone, co-ingestion of alkaline water and L-glutamine increased the salivary testosterone concentration after training, whereas no changes were noted when supplemented with either alkaline water or L-glutamine alone. Testosterone induces anabolic and anti-catabolic mechanisms involved in the growth, recovery, remodeling of muscle tissues, and performance enhancement. An augmentation of the testosterone response may be viewed as positive for enhancing recovery, given greater circulating testosterone levels and potential for tissue uptake and receptor binding [63]. Previous research has highlighted the association between low testosterone levels and increased risk of metabolic issues and systemic inflammation [64]. A past study has demonstrated that pro-inflammatory cytokines such as IL-6, tumor necrosis factor-alpha (TNF-α), and interleukin-1 beta (IL-1β) may inhibit testosterone secretion by modulating the hypothalamic–pituitary–gonadal axis [65]. Both L-glutamine and alkaline water have anti-inflammatory properties and are known to reduce inflammatory cytokines [66,67]. Accordingly, we speculate that these two supplements synergistically exerted anti-inflammatory effects and modulated the athletes’ physiological responses during intensified training sessions. Nevertheless, we cannot extend this speculation to cortisol, salivary lactoferrin, and IgA.

Concerning cortisol, it is the major human glucocorticoid hormone released in response to physical and psychological stress, is a strong suppressor of the immune response, and exhibits catabolic actions in the human body [68]. These actions are critical to the promotion of protein synthesis, which is necessary for the adaptation process in response to stressful situations [69]. During short-term exercise, cortisol levels increase in proportion to exercise intensity [70]. However, the hormone response tends to be attenuated after adaptation and physical improvement to the training program [69,71]. In this study, salivary cortisol levels were not changed regardless of whether alkaline water or L-glutamine was ingested alone or they were co-ingested after training, implying that the athletes were adapted to the training program. Another possible reason might be a delayed change in salivary cortisol. Post-exercise cortisol peaks occurred at 10–20 min in plasma and at ~30 min in saliva and plasma, whereas peak salivary testosterone occurred at 0–10 min [20], which might explain why we found a change of salivary testosterone but not cortisol.

Previous studies have shown that daily intake of 3~4 L of alkaline water for 3 weeks can improve combat sport athletes’ anaerobic performance in two double 30 s Wingate tests for the lower and upper limbs with a passive rest interval of 3 min between the bouts of exercise [28]. Drinking bicarbonate-rich water improved hypo-hydrated judo athletes’ buffering capacity and anaerobic performance [72]. Basketball players supplemented with 2.5~3 L of alkaline water for 6 weeks showed a positive impact on the acid–base balance of the body as well as on anaerobic performance in a 6 × 28 m shuttle run [29]. Drinking 4 L of mineral-based alkaline water for 7 days had a positive effect on urine pH and lactate utilization after supramaximal exercise in well-trained soccer players [73]. In addition, ingesting alkaline water for 5 months led to less severe gastritis and reduce inflammation [74]. An animal study found beneficial effects of alkaline water on aspirin-induced gastric mucosal injury and suggested that these effects may be attributed to its anti-inflammatory properties via inhibition of TNF-α expression [75].

The above-mentioned studies indicate that this supplement’s anti-acid and anti-inflammatory properties result in superior recovery for athletes after training or competition, which is important for athletes’ immunosurveillance and immunocompetence [76]. However, our experiment using acute and single doses of 400 mL of alkaline water did not show a beneficial response on salivary immune markers. In contrast, most previous studies adopted chronic supplementation, and assessed athletes’ performance and acid–base balance, not their immunity. There were no other studies investigating the acute or chronic effects of alkaline water on athletes’ immune functioning. The present study suggests that a single dose of alkaline water is insufficient to produce a significant change in salivary immune markers after exercise.

There are a number of limitations in the present study; the first is that we only designed the experiment for acute supplementation, and it is possible that long-term supplementation with these nutritional aids would affect the concentrations of salivary immune markers and hormone responses. Additionally, the dose of alkaline water in our experiment was 400 mL, which is far less than the average of 2.5–4 L/day reported in other studies. The reason we selected this dose was because the athletes had to finish the whole amount of the supplement immediately before training, and it is not practical for athletes to drink a huge amount of water before training.

Furthermore, the sample size of our participants was relatively small, and we only included male athletes in our study. Thus, the results cannot be conclusively extrapolated to female athletes. We speculate that these results might be applicable to other combat sports that utilize a similar energy system to boxing, such as taekwondo and karate [77]. However, we cannot extend this applicability to other sports such as endurance events. In the future, more studies recruiting larger numbers of athletes, including females and athletes from different sports, are needed in order to extend the applicability of this study.

## 5. Conclusions

In summary, our experiment demonstrated that co-ingestion of 400 mL alkaline water and 0.15 g/kg L-glutamine before training enhanced boxers’ salivary α-amylase activity and testosterone concentration. Acute supplementation with alkaline water or L-glutamine alone had no effect on salivary immune markers or salivary hormone response after training. The results of this study indicate the benefits of co-ingestion of alkaline water and L-glutamine on the salivary immunity and hormone status of boxers, which might be beneficial for boxing athletes’ recovery after training.

## Figures and Tables

**Table 1:** The characteristics of the boxing athletes (*n* = 12).

Parameter	Mean ± SD
Age (years)	22.42 ± 1.78
Height (cm)	174.42 ± 6.29
Weight (kg)	77.3 ± 13.5
Body mass index (kg/m^2^)	25.3 ± 3.1
Training years	8.19 ± 3.25
Weekly training hours	15.33 ± 1.15

**Table 2:** Physical activities, heart rates, and rates of perceived exertion of the participants.

Group	A	G	A+G
Physical activity (min)			
Sed	5.43 ± 5.17	4.90 ± 3.90	5.09 ± 4.80
Light	14.24 ± 9.68	12.66 ± 5.01	13.31 ± 8.61
MVPA	40.79 ± 13.98	42.44 ± 6.13	39.18 ± 13.34
Heart rate (beats/min)			
PRE	73.4 ± 10.8	72.8 ± 13.3	68.4 ± 7.6
POST	120.9 ± 12.8 ***	122.9 ± 17.2 ***	121.7 ± 12.4 ***
Rate of perceived exertion			
PRE	3.2 ± 2.0	2.7 ± 2.1	2.7 ± 1.7
POST	7.4 ± 1.6 ***	7.5 ± 1.4 ***	6.9 ± 1.7 ***

A: alkaline water group; G: L-glutamine group; A+G: alkaline water + L-glutamine group. Sed: sedentary; Light: light physical activity; MVPA: moderate to vigorous physical activity. Values are mean ± SD. *** *p* < 0.001, significantly different from PRE in each group.

**Table 3:** Effects of ingestion of alkaline water and L-glutamine on the ratio of salivary α-amylase activities/TP and the concentrations of salivary lactoferrin/TP and IgA/TP.

Group	A	G	A+G
α-Amylase/TP (U/mg)			
PRE	54.06 ± 18.64	59.83 ± 28.93	55.34 ± 33.62
POST	64.89 ± 21.65	66.32 ± 19.85	66.37 ± 37.29 *
Lactoferrin/TP (µg/mg)			
PRE	6.37 ± 3.50	5.98 ± 2.67	5.00 ± 2.17
POST	4.56 ± 2.03	5.92 ± 2.17	4.49 ± 2.62
IgA/TP (µg/mg)			
PRE	107.54 ± 42.29	107.93 ± 37.15	113.07 ± 54.03
POST	96.58 ± 36.95	100.25 ± 40.07	103.16 ± 50.23

TP, total protein; A, alkaline water group; G, L-glutamine group; A+G, alkaline water + L-glutamine group. Values are mean ± SD. * *p* < 0.05, significantly different from PRE in each group.

## Data Availability

Data are contained within the article.
